# Starch branching enzymes as putative determinants of postharvest quality in horticultural crops

**DOI:** 10.1186/s12870-021-03253-6

**Published:** 2021-10-21

**Authors:** Jingwei Yu, Keyun Wang, Diane M. Beckles

**Affiliations:** 1 Department of Plant Sciences, University of California, One Shields Avenue, Davis, CA 95616 USA; 2 Graduate Group of Horticulture & Agronomy, University of California, Davis, CA 95616 USA; 3 Present Address: Institute of Plant and Food Science, Department of Biology, School of Life Sciences, Southern University of Science and Technology, Shenzhen, 518055 PR China

**Keywords:** Starch branching enzyme, Horticultural crops, Postharvest quality, Postharvest shelf-life

## Abstract

Starch branching enzymes (SBEs) are key determinants of the structure and amount of the starch in plant organs, and as such, they have the capacity to influence plant growth, developmental, and fitness processes, and in addition, the industrial end-use of starch. However, little is known about the role of SBEs in determining starch structure-function relations in economically important horticultural crops such as fruit and leafy greens, many of which accumulate starch transiently. Further, a full understanding of the biological function of these types of starches is lacking. Because of this gap in knowledge, this minireview aims to provide an overview of SBEs in horticultural crops, to investigate the potential role of starch in determining postharvest quality. A systematic examination of SBE sequences in 43 diverse horticultural species, identified SBE1, 2 and 3 isoforms in all species examined except apple, olive, and *Brassicaceae*, which lacked SBE1, but had a duplicated SBE2. Among our findings after a comprehensive and critical review of published data, was that as apple, banana, and tomato fruits ripens, the ratio of the highly digestible amylopectin component of starch increases relative to the more digestion-resistant amylose fraction, with parallel increases in SBE2 transcription, fruit sugar content, and decreases in starch. It is tempting to speculate that during the ripening of these fruit when starch degradation occurs, there are rearrangements made to the structure of starch possibly via branching enzymes to increase starch digestibility to sugars. We propose that based on the known action of SBEs, and these observations, SBEs may affect produce quality, and shelf-life directly through starch accumulation, and indirectly, by altering sugar availability. Further studies where SBE activity is fine-tuned in these crops, can enrich our understanding of the role of starch across species and may improve horticulture postharvest quality.

**Supplementary Information:**

The online version contains supplementary material available at 10.1186/s12870-021-03253-6.

## Background

Horticulture likely originated 20,000 years ago [1]. There are over 100 species of horticultural crops [2], consisting of diverse fruits, vegetables, and tubers [3], many of which are of high economic value with enormous production volume worldwide [4]. The amounts of fruits, vegetables, and tubers produced in 2018 were 868, 1089, and 832 million tons respectively (FAO, 2019), and the increased demand from a growing, and affluent global population, is predicted to drive further expansion of horticultural output [5, 6]. Horticultural crops not only provide basic calories (e.g., tubers and roots), but also, are among the most crucial sources of fiber, organic acids, micro- and macro minerals, vitamins, and antioxidants in human diets [7, 8]. Healthy attributes, and a wide range of tastes, textures, and flavors make horticultural crops attractive [5].

Starch is critical to human society given its versatile uses [9]. Starch is the dominant energy source in the human diet, providing over 50% of our daily caloric needs [10]. In the food industry, starch is widely used as a thickener, stabilizer, lipid replacer, defoaming agent, gelling agent, emulsifier, and dietary fiber, and in the pharmaceutical industry, starch is used as an excipient for drug delivery [11–13]. In addition to these diverse uses, starch is an excellent renewable material for making ethanol biofuels and degradable ‘bioplastic’ products [14].

Starch is almost ubiquitous in higher plants [10, 15], including horticultural crops, in ways that may or may not be noticed. For instance, potato, sweet potato, yam, and cassava are starchy, but spinach, lettuce, and ripe tomatoes, berries, and citrus are not, yet starch is likely to be important to the growth, development and fitness of all of these crops, as they are in better studied models [16–19].

The widely accepted view is that starch accumulates either in a transitory state, or for long-term storage starch [20]. Transitory starch follows a diurnal pattern: it is synthesized and accumulated directly from the products of photosynthesis in the leaf and in the stem during the daytime, and is then degraded into sugars as an energy source for the following night [21]. In comparison, storage starch is defined as that located in perennating organs such as seeds, grain, embryos and tubers [15, 22], where it provides sustenance for the next generation during germination and sprouting in sexual and asexual propagated crops, respectively [23]. A third class of starch: ‘transitory-storage starch’ has been proposed [24, 25]. It describes starch that is accumulated and degraded during development in the storage organ [24, 25]. Transitory-storage starch is a feature of many species including horticultural crops of economic value such as tomato, banana, kiwi, strawberry, nectarine, and apple fruit [26, 27].

Starch accumulates as semi-crystalline, water insoluble granules that vary in diameter from 1 to 100 μm depending on species [15]. Starch is organized into two glucan polymers: amylose and amylopectin [28]. Amylose and amylopectin consist primarily of linear chains of glucoses joined by α-1,4-glycosidic bonds [29]. In amylopectin, the α-1,4-glucan chains are branched more frequently (~ 5% of the linear chains) through α-1,6-glycosidic bonds, compared to amylose [29]. The branching of the amylopectin chains is such that chains of different lengths are produced: short, medium and long chains, and the frequency with which each fraction occurs influences starch functionality [30]. Side chains of amylopectin form clusters around branching points, and two adjacent chains make up a double helix [31]. These physical features of amylopectin polymers leads to a semi-crystalline granule; amylose with a randomly coiled conformation, fills the matrices within the granule [32]. Amylopectin and amylose account for around 25 and 75% of the starch in major heterotrophic storage organs, respectively [33], while the starch in leaf tissues is approximately 5 - 10% amylose [34].

## Main text

Amylose and amylopectin are synthesized by the coordinate action of a group of four key enzymes [35]. The core starch biosynthetic enzymes include ADP-glucose pyrophosphorylases (AGPases), starch synthases (SSs, granule bound or soluble), starch branching enzymes (SBEs), and de-branching enzymes (DBEs), of which there are many isoforms [36]. In brief, AGPases initiate the first step of starch biosynthesis by catalyzing the formation of ADP-glucose [37, 38]. SSs elongate the glucan chains in amylose and amylopectin [39]; SBEs branch the glucan chains [40], while the DBEs shorten and modify the starch chains which enable a higher-order semicrystalline structure to form [41]. SBEs, the focus of this review, hydrolyze α-1,4-linked glucan chains, and attach the newly-created ‘free’ chain to another glucan chain within the starch granule, via an α-1,6-linkage (Figure S1) [42]. Through this action, SBEs largely determine the proportion of the relatively unbranched amylose to the highly-branched amylopectin [40, 43, 44].

Two major classes of SBEs are biofunctionally known: SBE1 and SBE2 (Table 1), and they vary in terms of their substrate selectivity [40], whereas the function of SBE3 awaits verification across a broader set of species. SBE1 preferentially branches ‘amylose-like’ long glucan chains as the substrate, while SBE2 prefers a more branched substrate [10]. The action of both forms further increases the number of branch points in starch polymers [40].

**Table 1:** Examples of Starch Branching Enzyme (SBE) nomenclature

**Species**	**Names used in this paper**	**Other names reported**	**Class**	**Locus tag/Gene symbol**
Maize (*Z. mays*)	SBE1	SBEI	1	LOC542315
	SBE2a	SBEIIa	2	LOC542342
	SBE2b	SBEIIb	2	LOC542238
Potato (*S. tuberosum*)	SBE1	SBEB	1	LOC102596498
	SBE2	SBEA	2	LOC102590711
	SBE3	SBE3	3	LOC102603708
*Arabidopsis thaliana*	SBE3	BE1	3	AT3G20440
	SBE2.1	BE3/BE2.1	2	AT2G36390
	SBE2.2	BE2/BE2.2	2	AT5G03650

SBEs are the key players in the regulation of the amylose-to-amylopectin proportion in plants. However, their functions in many harvested horticultural crops have been under-investigated, although evidence points to the importance of starch in determining the postharvest quality of these crops. We aimed to develop a better understanding of the role of SBEs in fruits, tubers, and leafy greens in physiological processes by exploring SBE sequence relationships, expression, and starch phenotypes in diverse crops.

### Cereal SBEs diverge from the majority of horticultural SBEs

**Fig. 1:**
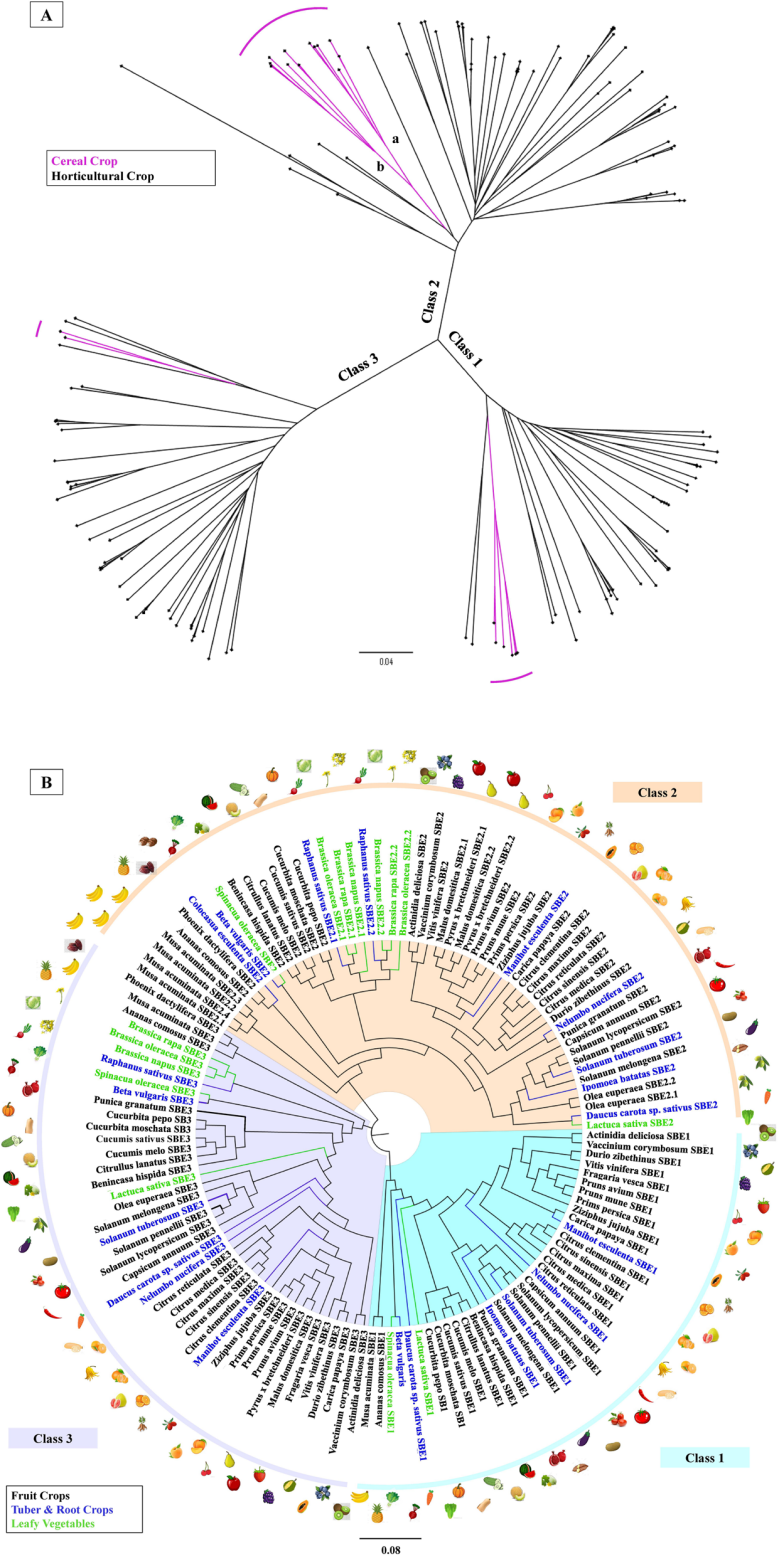
The three starch branching enzymes (SBEs) clades. **A**) A phylogenetic tree based on SBE DNA sequences from cereal and horticultural crops. The three clades correspond to the three classes of SBE, i.e., SBE1, SBE2, and SBE3. Cereals and horticultural crops diverged in each class. Within the class 2 SBEs, cereals form two clades, representing the ‘a’ and ‘b’ sub-isoforms (See Table 1). Only a few members of the predicted SBE3s were retrieved from cereals. This tree includes species from: rice, wheat, barley, sorghum, corn, millet, apple, banana, blueberry, rapeseed, cabbage, bok choy, citruses, cucumber, wax gourd, muskmelon, watermelon, pumpkins, date palm, durian, apricot, jujube, kiwifruit, lettuce, olive, papaya, peach, pear, pineapple, tomato, potato, pepper, eggplant, spinach, strawberry, sweet cherry, carrot, cassava, lotus root, radish, sweet potato, taro, and table grapes. **B**) A phylogenetic tree based on the predicted amino acid sequence of various SBE genes identified from horticultural crops showing sequence divergence. SBE1 evolved earlier than SBE2 and SBE3. SBE1 and SBE2 are more homologous to each other than to SBE3. SBEs from fruits, tuber & root, and leafy green were highlighted accordingly. SBE1 is absent in crops from the *Brassicaceae* family, apple, and European olive, while these species have two types of SBE2. Species presented include apple, banana, blueberry, rapeseed, wild cabbage, mustard, citruses, cucumber, wax gourd, muskmelon, watermelon, pumpkins, date palm, durian, apricot, jujube, kiwifruit, lettuce, olive, papaya, peach, pear, pineapple, tomato, potato, pepper, eggplant, spinach, strawberry, sweet cherry, carrot, cassava, lotus root, radish, sweet potato, taro, and table grapes. Sequences were retrieved from NCBI, Mainlab Bioinformatics Program (WSU) [52, 53] Sol Genomics Network [54], Genome Database for Vaccinium [55], CuGenDB [56, 57], Pineapple Genomics Database [58, 59] SpinachBase [60], KEGG [61], and Ensembl plants [62]. This tree was built by using the Neighbor-joining method with genetic distance (Jukes-Cantor Model) in the Geneious Prime® (Version 2020.2, https://www.geneious.com). The bootstrap test was performed with 1000 replicates. The figure generated was annotated using Microsoft® PowerPoint

SBEs have three classes of isozymes including two functional SBE classes (SBE1 and SBE2) and one putative class 3 SBE (Fig. 1A). SBE1 isoforms appeared earlier than SBE2 and SBE3 in the viridiplantae, but plant SBE1 and SBE2 are more homologous to each other, than to SBE3 [45–47]. SBEs have been identified and relatively well-characterized in cereal crops, tubers, and *Arabidopsis thaliana* over the last two decades [48–51], but, as mentioned, little attention has been paid to the diverse group of species that are classified as horticultural crops.

Within each class of SBE, the cereals grouped together, while most non-cereals formed another cluster (Fig. 1A). This pattern is due to the divergence of monocots from dicots around 200 million years ago [46]. In contrast to the presence of ‘a’ and ‘b’ sub-isoforms of SBE2 in cereal crops [63], horticultural plant species generally have one SBE2 isoform. It was also observed that not all species have a known or predicted class 3 isoform.

The SBE sequences contained within diverse organs, i.e., fruits, tubers, roots, and leafy vegetables (Fig. 1B), clustered together based on their respective plant families. The class 1 SBE is absent in *Arabidopsis thaliana* [28], and so it was not surprising that this SBE class is not present in the *Brassicaceae*. However, the class 1 SBE is also absent in apple (*Malus*), and European olive (*Olea*), but these species all have two class 2 SBE isoforms (Fig. 1B). In addition, banana contains at least four types of SBE2, and transcripts corresponding to these SBE2s have been identified, indicating that they are expressed [64].

### The domain features of SBE1 and SBE2 are highly conserved while those of SBE3 are not

**Fig. 2:**
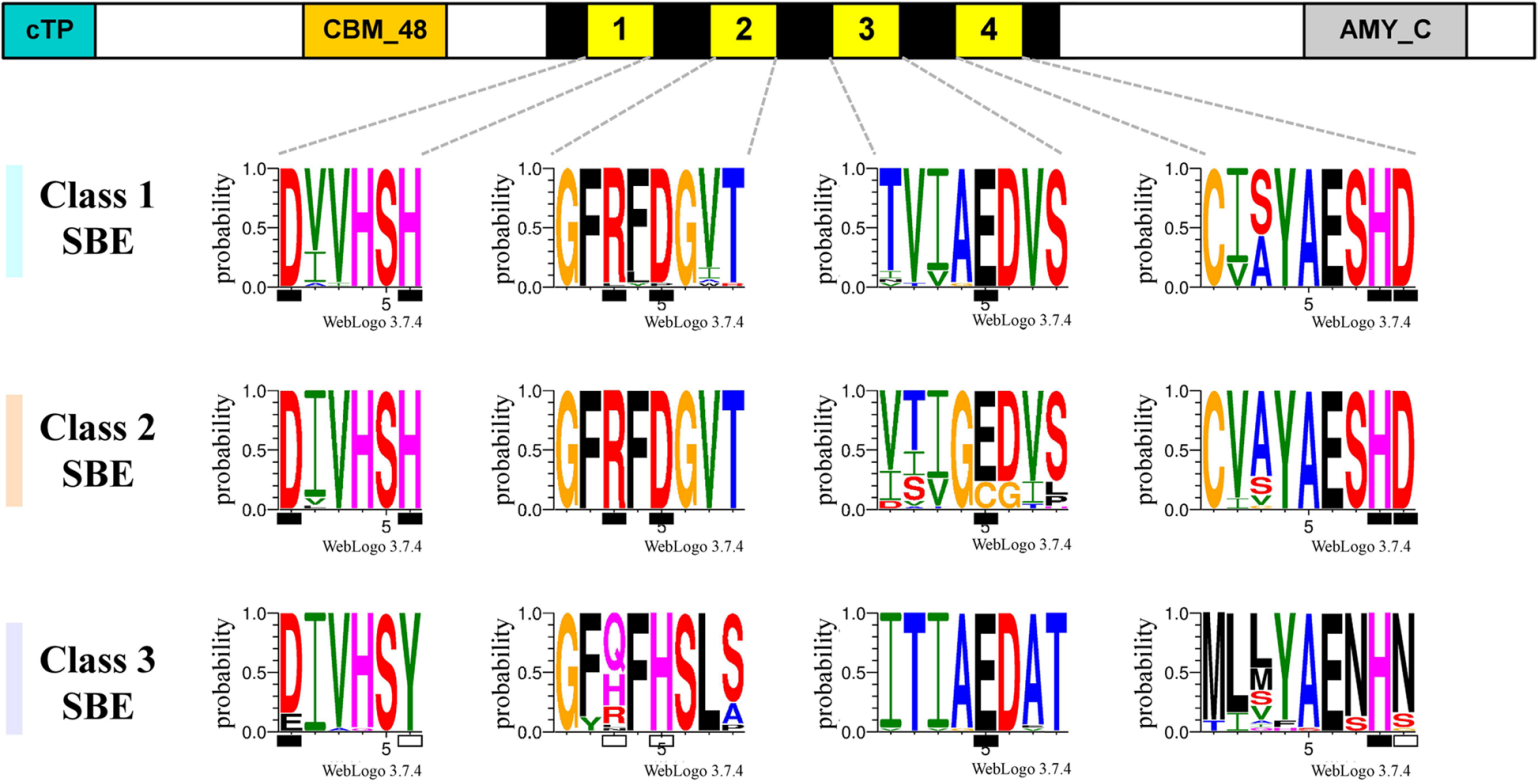
Critical regions in the predicted amino acid sequence of starch branching enzymes (SBEs) in the catalytic A domain in horticultural crops. The conserved SBE1 and 2 residues are invariant but the residues in the SBE3 isoform contain many substitutions. The four critical regions located within the central A-catalytic domain (black area in the middle of SBE protein) were assigned as Regions 1 to 4, respectively. The Regions within the SBE3 are less conserved than those in SBE1 and SBE2. A chloroplast transit peptide (cTP), a carbohydrate-binding module family 48 domain (CBM_48), and an α-amylase C-terminus (AMY_C) are shown. The small black bars on the x-axis indicate the catalytic/active residues, while the white bars represent variant residues at those sites. The Y-axis of each logo shows the probability of residues present on that specific site from the species listed in the Fig. 1. Residue logos were generated from WebLogo3 [70], and the figure was made in Microsoft® PowerPoint

Starch Branching Enzymes (E.C. 2.4.1.18) belong to the α-amylase family of enzymes, specifically the glycoside hydrolase family 13 superfamily [65, 66], with multiple isoforms encoded by different genes (Figure S2). The overall structure of the SBE polypeptide is highly conserved [67]: all SBEs possess a central α-amylase catalytic domain (the A domain), and an NH_2_- terminus, and a carboxyl- terminus (Fig. 2, S3) [68, 69].

The SBE NH2-terminus contains two conserved domains: a chloroplast transit peptide for plastid-targeting, and a CBM48 (carbohydrate-binding module 48) domain for binding to starch [71]. The C-terminus contains the residues that determine substrate preference and catalytic activity [40]. The central region of the enzyme contains the “A” catalytic domain, that is made up of 8-(β/α)-barrels [68]. Notably, the class 3 SBE may not directly participate in starch biosynthesis in *Arabidopsis* [49, 72], but it has a demonstrated function in mediating cesium toxicity of photosynthesis [73]. However, the role of SBE3 is unlikely to be conserved. In potato, StSBE3 has a unique coiled-coil motif which is absent in the AtSBE3 polypeptide (Figure S3). Notably, the CBM48 domain is also deficient in AtSBE3 (Figure S3). It is possible that the StSBE3 may interact and complex with other starch biosynthetic enzymes through its coiled-coil domain, in a similar way to the SS4-PTST2 interaction in *Arabidopsis* [74], the GBSS-PTST1 interaction in rice [75] or the SBE-containing protein complexes in cereal endosperm [76], rendering an assistant function in starch biosynthesis. This species-specific mode of action of SBE3 may reveal a novel function of SBEs generally. Indeed, although all SBEs are predicted to form complexes with starch phosphorylases (PHO1 and PHO2), the starch synthases (GBSS, SS1 or SS4) and isoamylase (ISA) (Table S2; Figure S5), interactions with other proteins show differences depending on the species and SBE isoform.

### The SBE3 group lacks the conserved residues in the A-domain critical for catalysis

Four conserved regions critical for catalysis, named Regions 1-4 (Fig. 2), are found within the catalytic A-domain (reviewed by Tetlow and Emes [40]). Regions 1-3 are directly involved in catalysis, while Region 4 is involved in direct substrate binding (Fig. 2) [67]. SBE1 and 2 have largely invariant residues, but the residues in the SBE3 isoform of many species have substitutions at these sites. Post-transcriptional phosphorylation of the SBE-protein complexes formed with other starch biosynthetic enzymes has been found in cereal crops and in cassava [76–79], while experimental evidence of this regulation in the majority of horticultural crops is absent. SBE1 and SBE3 have fewer possible phosphorylation amino acid sites than SBE2 (data not shown). Overall, the distinctive domain features of the SBE3 predicted protein, and the implifications for functionality may complicate current views of SBE function, but these features may also provide an opportunity to deepen our mechanistic understanding of starch biosynthesis and regulation.

### The SBE gene family contains cis-elements that indicate gene activation by environmental signals and hormones

Starch metabolism is tightly regulated by plants’ internal clock and the external day-night shifts, especially in photosynthetic organs where transitory starch turnover occurs on a daily basis [80]. The transcriptional response of the SBE genes follows the circadian rhythm in photosynthetic, and, in some cases, storage tissues [81]. Cis-elements related to circadian control and light responsiveness were universally present in all the horticultural SBEs examined (Figure S4). Hormones, such as abscisic acid (ABA), ethylene, salicylic acid (SA), jasmonic acid (MeJA), and sugar signals have been reported to regulate SBE activity in cereal and horticulture crops [81–83]. In addition, transcription factors (TFs) that belong to the WRKY, MYB, bZIP, AP2/EREBP families, may bind to their cognate cis-elements in the 5′ upstream regions of SBEs to activate or suppress transcription [64, 84–86]. However, information on the transcriptional regulations of SBE is fragmented, and putative hub genes or master regulators have not been identified [87]. System-wide surveys of cis-elements and TFs in combination with in vitro and in vivo experiments could shed light on, and unearth such regulatory networks.

### Amylose-to-amylopectin ratio in horticultural starches: end-use and functionality

The amylose-to-amylopectin ratio influences the textural, cooking, and nutritional properties of starchy foods, and the functionality of starch-derived biomaterials [9, 88–92]. Most of this structure-function analysis has been performed on starches isolated from cereals and tubers [32]. However, the relative proportions, and molecular structure of amylose and amylopectin in unripe fruit may have unique properties that could have specialized applications distinct from these well-characterized starches [93]. There may be additional markets for fruit starches if premature harvest occurs, or is desirable, due to climactic events [24].

Starch, or the proportion of the amylose fraction of starch, is used as a common ripening biomarker for apple [94], banana [95, 96], and pear [97]. This marker relies on the ability of amylose to physically interact with iodide to form a triiodide blue-black complex.

Starch can also influence the quality of fruit juice. Although starch is degraded to sugars when fruit ripens, this conversion is not complete. Ripe fruit processed for juice therefore contains starch, which is treated with amylases for clarification [98]. Further, the amylose content of the remanant starch in some fruit processed for juice, may alter juice viscosity [99].

## Putative role of SBEs as determinants of postharvest quality in horticultural crops

### Deducing SBE function in leafy greens using Arabidopsis rosettes as a model

**Fig. 3:**
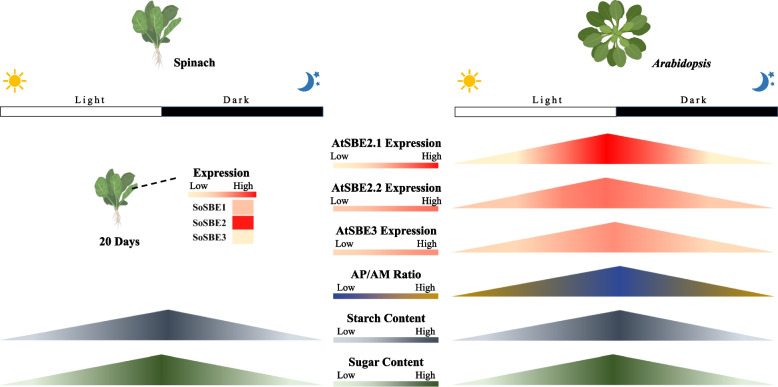
Transitory starch and starch branching enzymes (SBEs) dynamics in spinach and *Arabidopsis* leaf summarized from existing publications. In *Arabidopsis* rosettes, the pattern of net accumulation of starch, sugar, and SBE transcripts over the day-night transition are similar. In spinach, diurnal starch and sugar level changes are similar to *Arabidopsis*. SBE transcriptional profiles are unknown in spinach. The starch and sugar diurnal changes in spinach with data adapted from [108], and the right upper panel shows the SBE mRNA level, amylopectin-to-mylose ratio (AP/AM), and starch and sugar daily dynamics [109, 110]. Spinach SBE (SoSBE1, Spo04764; SoSBE2, Spo06399; SoSBE3, Spo09493) expression data were obtained from SpinachBase [60]. Graphs were drawn based on published data found in Table S1 using Microsoft® PowerPoint

Prepackaged leafy greens are convenient and healthy, and are popular options for salads in western countries [5, 100]. Metabolism in this horticultural product can be considered over distinct phases in its lifecycle: pre- and postharvest [101]. In developing spinach, the photosynthetic organ, i.e., the leaf, fixes carbon, and partitions a large portion ~ 20% to starch biosynthesis during the light period under lab conditions [102]. Starch accumulates linearly across the daytime at an almost constant rate (paralleling an increase in sugar content) (Fig. 3). During the night, the leaf starch is degraded into sugar, to maintain plant metabolism, resulting in an empty polysaccharide reserve before the next light period [103, 104]. In *Arabidopsis*, the expression of SBEs and the changes of amylopectin and amylose (AP/AM) show a similar trend, but there is variation in when SBE transcripts peak. Although there is no information on SBE transcriptional levels in spinach during the diel, there may be some similarities with *Arabidopsis* because the pattern of leaf starch accumulation is comparable in spinach and *Arabidopsis* [105–108].

**Fig. 4:**
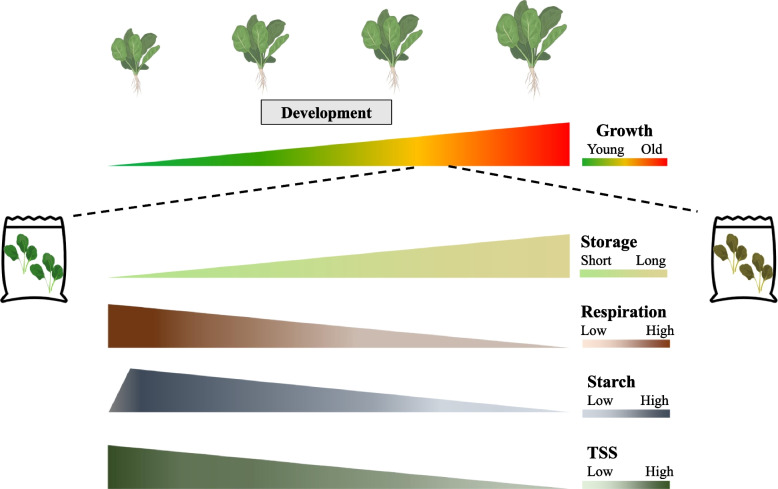
Starch and sugar dynamics in postharvest spinach leaf summarized from existing publications. Starch and sugar content decreased during the storage of harvested spinach leaves. The lower panel presents the respiratory activity, starch, and sugar content as TSS, i.e., total soluble solids, in postharvest packaged spinach [122, 123]. Graphs were drawn in Microsoft® PowerPoint using the published data found in Table S1

The preharvest starch reserve may alter the postharvest quality of leafy greens. Harvested green produce are stored in optimized packaging under limited light exposure conditions which restricts new energy and carbon input from photosynthesis (Fig. 4) [111–113]. However, respiratory activity, which is the carbon skeleton generation process for cellular metabolites, although reduced, does not stop [26]. In detached leaves, the starch can be broken down to glucose, and sugars become the main source of fuel for cellular metabolism and ATP generation in the early stage of respiration [111, 114, 115]. In the late stage of the respiratory process, the depleted sugars will be replaced by proteins, lipids, and membranes, triggering leaf senescence and cell death [26, 116]. This results in undesirable produce quality and ultimately, in produce loss [5]. Preharvest and postharvest starch content may determine postharvest energy reserves and influence the timespan that buffers the onset of senescence, thus influencing shelf-life of harvested green leaves [114, 117, 118]. Correlations between leaf starch content and postharvest longevity have been found. For example, lettuce and red chard harvested at the end of the day, when leaf starch content was highest, had a longer extended shelf-life than organs harvested at other times of day [114, 117, 118]. This may not be true of all varieties e.g., salad roquette [117]. Starch also correlated with improved shelf-life quality after light exposure to detached leaves in vegetables such as Chinese kale and lettuce [118–121]. The accessibility of sugars from the degraded starch may relate to leafy-green quality, and the upregulation of SBEs would convert amylose to the more catabolically available amylopectin, providing a more readily available source of sugar.

The amylose-to-amylopectin ratio in *Arabidopsis* influences flowering time and reproductive growth, key markers of development, and fitness [23, 124, 125]. Whether starch molecular structure and composition influences the preharvest growth of leafy greens in a similar way, remains unknown, but it seems likely.

### SBEs are determinants of potato and cassava postharvest tuber quality

**Fig. 5:**
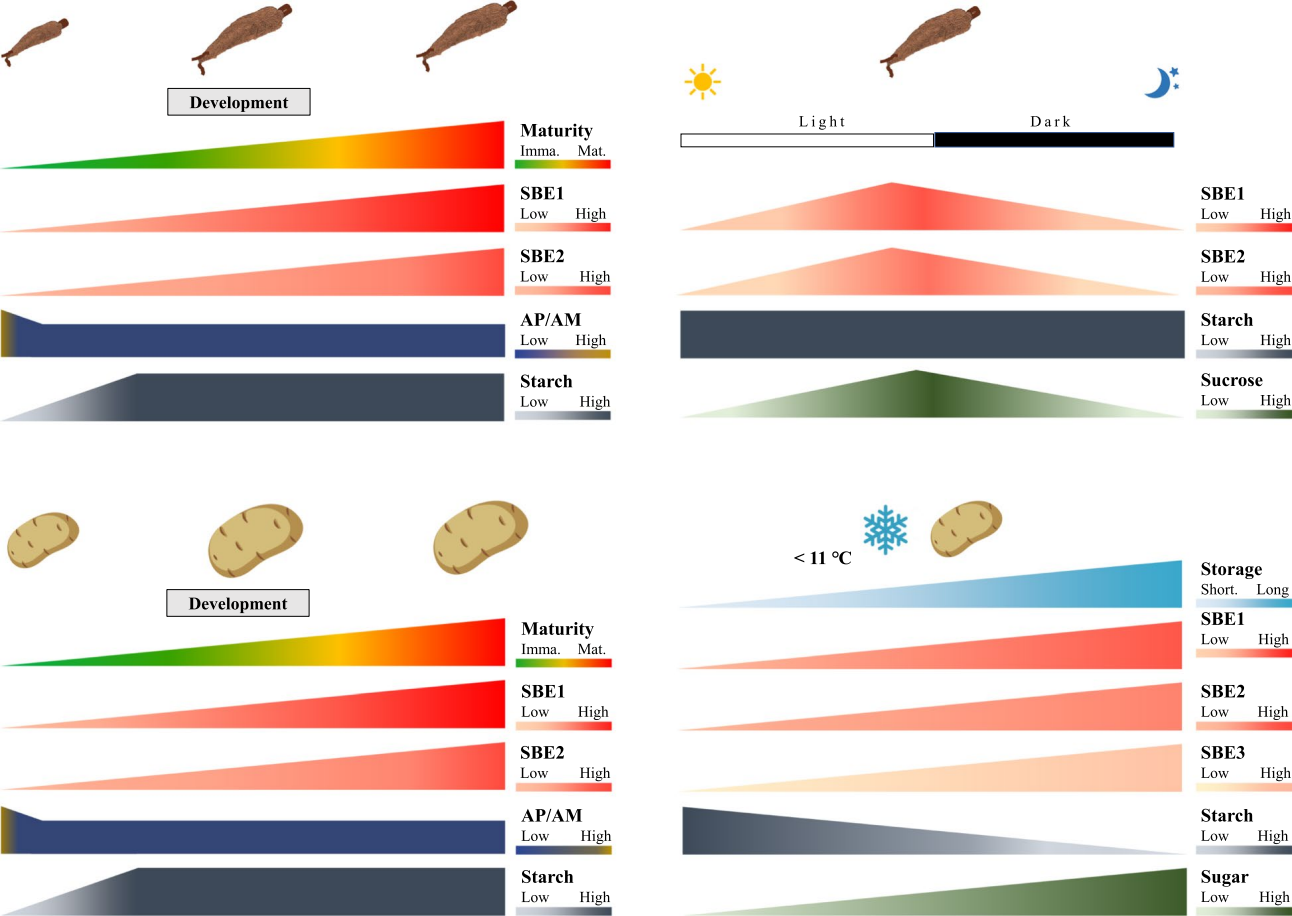
Changes in starch, and starch branching enzyme (SBE) transcripts in cassava root and potato tuber across storage organ development, the diurnal cycle (cassava) and during cold storage (potato). SBE transcriptional changes paralleled changes in the amylopectin to amylose ratio in developing cassava roots, while the amylopectin-to-amylose ratio remains constant during potato tuber development. Cassava root starch quantity and quality, sugar content, and relative SBE expression were summarized from four publications [81, 137, 143, 144]. Potato tuber starch content and composition, relative SBE transcript expression during tuber development were adapted from [145, 146], and the cold storage changes were adapted from [147]. Graphs were drawn in Microsoft® PowerPoint using the published data found in Table S1

Potato, sweet potato, and cassava are generally considered as high glycemic index (GI) foods because the starch in their storage organs is easily digested to sugars when consumed, leading to a rapid increase in blood sugar level [126]. It is established that high GI food exacerbate metabolic disorders such as diabetes and obesity [127]. In contrast, multidisciplinary experimental research shows that digestion-resistant starch could increase the healthful microbial communities of the gastrointestinal tract, reducing the occurrence of constipation, and lowering the risk of colon cancer [90, 128]. Altering potato starch composition is a viable way to increase ‘dietary fiber’ content and to enhance colonic health. This can be achieved by either physical, chemical, or enzymatic modifications of purified starch, e.g., etherification, esterification, or by fine-tuning the activity of starch biosynthetic enzymes [129, 130]. Reduction or knockout of SBEs in a range of species have reliably led to an increase in the resistant starch (RS) content in various species including horticultural crops e.g., potato, sweet potato, and cassava, [75, 78, 130–140]. Interestingly, SBE2 is not the dominant isoform expressed in storage tubers and roots, but it exerts a major function in amylopectin synthesis [141]. Very high levels of RS can be achieved by the combined suppression of SBE1 and SBE2, but with a yield penalty [142]. The transcriptional profiles and functions of SBE3 are unclear in the developing tubers (Fig. 5).

In addition, potato tubers suffer from a postharvest disorder: cold-induced sweetening (CIS). Potato tubers are stored at low temperatures (< 11 °C) to extend shelf-life and to meet year-round demand [145]. However, sugars accumulate from starch breakdown, a process referred to as CIS (Fig. 5) [148, 149]. Although a problem for the potato industry, CIS could be a mechanism to allow tubers to cope with chilling stress [16, 18]. CIS negatively affects the quality of fried or baked potato products: reducing sugars react with free amino acids at high temperature cooking through the Maillard reaction, to form carcinogenic acrylamide [150, 151]. Changes in the enzymes involved in starch biosynthesis and degradation are involved in CIS [152]. SBEs are actively expressed in CIS susceptible tubers [147], and in *StVInv*-silenced, CIS-resistant tubers, SBEs transcriptional level were suppressed [153]. Naturally occurring high RS potato varieties, also, have less susceptibility to CIS [154]. Therefore, evidence points to a positive association of SBE activity with CIS severity in some potato genotypes.

### Ignored ‘transitory-storage starch’ may contribute to fruit quality

Starch is a major component of the dry mass of fruits at commercial harvesting time. Starch is transiently synthesized and stored in unripe fruits with a peak just before ripening [155]. Starch appears to be a critical feature of climacteric fruit metabolism, known for their bursts of respiratory activity and ethylene production upon ripening [27, 156]. Climacteric fruits contain more starch, and, more active starch biosynthesis than non-climacteric fruit after anthesis [27, 156]. In tomato, the functional genomics model for fleshy climacteric fruit, starch fulfilled 40% of the carbon needed for respiratory processes based on a constraint-based flux model [157]. Experimental evidence from postharvest metabolism also supports the model: tomato fruits stored postharvest under low or chilling temperatures undergo bursts of stress-related carbon dioxide and ethylene production when allowed to recover at room temperature, with an accompanying and corresponding decrease in starch reserves [158, 159]. A similar inverse relationship between starch content and respiratory activity was observed in ripening banana [96, 160–162], ginger rhizomes [163] sunberry [164], apple [165] and durian [166] (Figure S6; Table S3). The relationship between tissue starch content and respiration may not be perfectly linear in all species, e.g., in stored ginger, starch showed a biphasic accumulation pattern as respiration progressed, a trend not seen in other tissues examined (Figure S6; Table S3). Furthermore, the relationship between these variables may also differ among genotypes within a species.

**Fig. 6:**
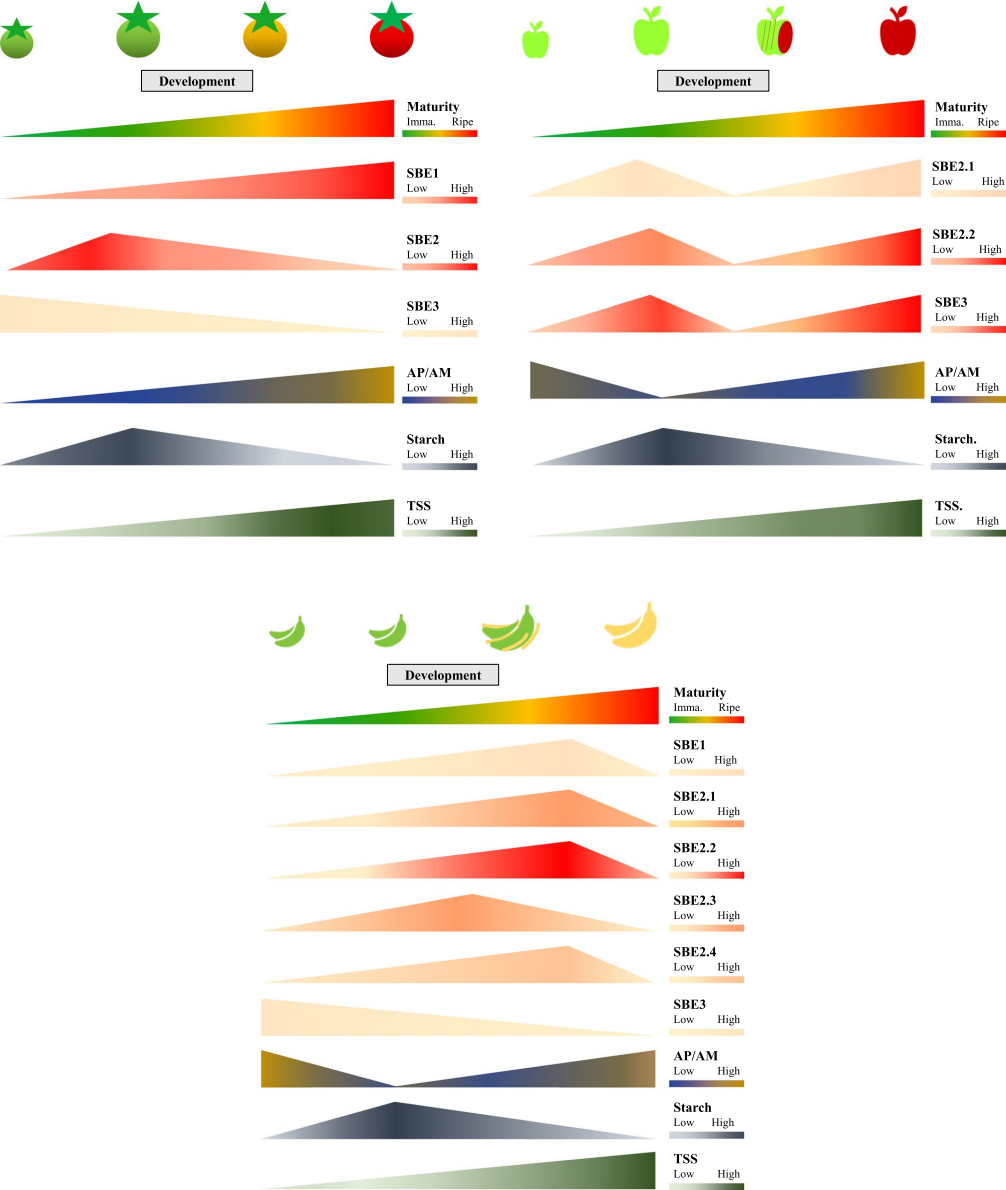
‘Transitory-storage starch’ and relative starch branching enzymes (SBEs) gene expression in developing and ripening fruits. SBE expression patterns in apple differ from that in tomato and banana, in that they distinctly shows bimodal peaks. In addition, unlike the other fruit SBE3s which decrease in expression, the apple SBE3, increases during fruit ripening. The starch content and changes in amylopectin-to-amylose ratio are similar in tomato, apple, and banana. Tomato SBE genes (SlSBE1, Solyc04g082400; SlSBE2, Solyc09g009190; SlSBE3, Solyc07g064830) expressions were obtained from BAR eFP [171], and carbohydrate contents were adapted from [169]. Relative expression level of apple SBE genes (MdSBE2.1, MD12G1020600; MdSBE2.2, MD14G1017700; MdSBE3, MD08G1002300) were retrieved from AppleMDO [172], the starch and sugar data were adapted from two publications [173, 174]. Banana starch and SBEs profiles were summarized from three publications [64, 161, 175]. TSS – Total soluble solids. Graphs were drawn in Microsoft® PowerPoint based on published data in Table S1

Apart from climacteric characteristics, after the onset of ripening, starch content plummets sharply accompanied by starch decomposition into soluble sugars, and the total soluble sugar content continues to rise proportionally (Fig. 6). This dynamic metabolic process had been reported for both climacteric and non-climacteric species including tomato, apple, banana, plantain, mango, kiwifruit, pear, and strawberry [27, 97, 155, 167–169]. Adequate storage of the starch-derived soluble sugars, is essential to produce an acceptably flavored horticultural produce of appropriate sweetness [170].

Accompanying the starch-sugar dynamics, amylopectin-to-amylose ratio (AP/AM), also changes interactively (Fig. 6). The difference in the AP/AM ratio in fruit development is expected to influence the structure of starch and its degradability. In the ripening tomato, the rate of decrease of amylose was greater than that for amylopectin [169]. Thus, the AP/AM ratio increased dramatically during ripening, in concert with the increase in soluble sugar content and fruit color change from green to red [169]. This phenomenon where the proportion of amylopectin increases relative to amylose, was also evident in ripening apple and banana [64, 173]. It is possible to speculate that of the available starch left during fruit ripening, the amylose, or long-chained amylopectin was converted into amylopectin whose branch-like structure has a much higher susceptibility to enzyme attack, allowing the rapid process of starch degradation into soluble sugars and supply for respiration. However, this mechanism may not be universal for all fruit. For example, the changes in AP/AM ratio in kiwifruit are similar to those in developing potato tubers, where the ratio of AP/AM almost remains constant during tuber development (Fig. 5) [155]. In ripening tomato fruit with sharp increases in AP/AM, up-regulation of SBEs transcriptional expression is expected. Among SBEs, the class 2 SBE has the major effect on altering starch compositions [40, 141]. Elevated expression of SBE2 transcripts does parallel the changes in the AP/AM in ripening tomato, apples, and banana. We propose that ultimately, this change in glucan structure indirectly contributes to flavor, quality, and commodity value.

## Altering the postharvest quality of horticultural produce by modifying starch

Starch, in general, plays an essential role in balancing the plant’s carbon budget as a reserve of glucose that is tightly related to sucrose metabolism and sugar signaling pathways [23]. Starch is considered as an integrative mediator throughout the plant life cycle, regulating plant vegetative growth, reproductive growth, maturation and senescence, and response to abiotic stresses [16, 18, 19]. This comprehensive regulation is achieved by changes in the synthesis and degradation of starch to balance glucose levels, after developmental and environmental triggers in different organs [176].

Transitory starch and its biosynthesis have been well studied in the model plant *Arabidopsis*, but little research has been conducted on postharvest leafy greens. Quality metrics such as shelf-life, flavor, color, firmness, and texture are of consumers’ choice, and they are related to the limited pools of storage compounds in detached leaves, which cells rely on to maintain basic cellular activities [26]. A hypothesized function for the starch in packaged leaves could be presented as such: starch may act as a buffer against sugar starvation, and protect against cellular autophagy, by serving as an alternative energy source [103]. If the biosynthesis and degradation of starch could be adjusted in a controlled way, then the modulated release of sugars may influence the postharvest shelf-life in detached leafy greens (Fig. 4). A continuous, paced supply of sugars may preserve vacuolar nutrients and water content, leaf cellular structure and integrity, and, thus extend the ‘best by’ postharvest date of the produce. Although the eco-physiological role of amylose is poorly understood in *Arabidopsis* [177], the AP/AM ratio may set a threshold for the optimum usage of starch. SBE action in leafy crops may differ from those in *Arabidopsis* given the dissimilar numbers of their isoforms and domain features (Figure S3). Modifying the quantity and quality of the starch in leafy greens such as spinach, lettuce, and watercress, by targeting starch biosynthetic enzymes, may provide evidence to its postharvest function in terms of produce longevity.

Resistant starch is a popular nutritional additive to produce food with enhanced quality attributes, i.e., higher fiber content, and starchy horticultural commodities are similarly attractive [128]. The yield penalty of high amylose crops may be alleviated by picking an ideal AP/AM ratio through a coordinate change in the relative balance of starch biosynthetic enzymes [44]. In the case of potato, it is plausible that downregulation of SBEs not only produces healthy fiber-starch, but also lessens the CIS severity and acrylamide problem (Fig. 5). However, the sugars derived from starch during CIS may be an adaptive mechanism to enhance plant chilling tolerance [16, 18]. Rapid sugar accumulation upon cold stress have been reported in fruit [158, 159, 178]. The sugars freed from starch may promote metabolic activity and serve as an osmoprotectant, thus alleviating chilling injury. The major functional SBEs were found to be upregulated in cold-stressed banana fruit, potato tuber, and *Arabidopsis* leaf [64], which may facilitate the ‘sugaring’ process. Modulating SBE activities may alter the rate of sugar released from the highly digestible starch polymers, thus changing the fruit/tuber cold responses.

In fruiting species, the importance of ‘transitory-storage starch’ may be underestimated due to the lack of enough direct knowledge of its function, gained from experimental data. Tomato serves as a functional genomics model for fleshy fruit, as it is easily transformed and genetically manipulated [179]. The putative function of ‘transitory-storage starch’ in fruit ripening, respiration, and sweetness enhancement may be revealed by engineering AP/AM ratio through overexpression or suppression of SBEs. We hypothesize that high amylose, resistant starch tomato fruit may have reduced available starch, sugars, and changes in fruit ripening and other processes that are dependent on starch as a carbon supply and source of energy postharvest. Tomato SBEs may not reflect the functionality of all fruit SBEs, but it would produce fundamental knowledge and expand our understanding of species-, organ- and developmental-specific regulations of the core starch biosynthetic enzymes.

## Conclusion

Numerous studies on *Arabidopsis* and cereal crops have advanced our understanding of starch biosynthesis in leaf and endosperm, and this knowledge has been applied to starch quality improvement in agronomical crops. On the contrary, the functions of starch in diverse horticultural crops are poorly understood, but it may play an essential role in their postharvest quality.

SBEs largely determine starch composition and function (Figure S1), and there are three major classes of SBEs across cereal and horticultural crops (Fig. 1A). Compared to the well-studied SBE1 and SBE2, the function of the emerging SBE3 isoform in horticultural crops remains unknown (Fig. 1B). Although SBE3 has less invariant catalytic residues compared to SBE1 and SBE2 (Fig. 2), the gene structure of the SBE3 is highly conserved (Figure S2) and as is the protein secondary structure, including the critical CBM48 module (Figure S3). A unique coiled-coil region may provide SBE3 with a distinctive role in starch metabolism as an ‘accessory protein’ through forming protein complexes with core starch biosynthetic enzymes.

SBEs in leafy greens, tubers and roots, and fruits show divergent transcriptional patterns during organ development (Figs. 4, 5, and 6). The activity of SBEs may influence postharvest quality of these crops, influencing starch digestibility to sugars and hence its ability to serve as an energy source during storage, thereby affecting shelf-life. The proportion of sugars affects tissue osmotic properties, and if sugars levels are optimal at the crucial stage of postharvest life, this may reduce wilting, thereby boosting the visual appeal of leafy greens. Upon consumption, the proportion of sugars available in fruit vs. that used for respiration, or that remaining as starch, could influence taste, i.e., sweetness and nutritional attributes. Therefore, modulation of SBEs in major edible organs of these produces could test these hypotheses, and broaden our understanding of tissue- and species-specific starch metabolism, and potentially improve the postharvest attributes of several horticultural crops.

### Supplementary Information

**Additional file 1: Figure S1.** Mode of action of the starch branching enzymes (SBEs). **Figure S2.** Starch branching enzyme (SBE) gene structure in select horticultural crops. **Figure S3.** Protein domain of starch branching enzymes (SBEs) in select horticultural crops and *Arabidopsis thaliana*. **Figure S4.** Predicted cis-elements of the 2 Kb upstream region of the SBE coding sequences. **Figure S5.** Predicted protein-protein interaction ‘STRING’ networks of selected SBE proteins. **Figure S6.** Correlation between starch content and respiration in diverse ripening produce.

**Additional file 2: Table S1.** SBE expression, starch and sugar content, relative levels of amylopectin (AP) and amylose (AM) in various species. **Table S2.** STRING analyses of potential SBE-protein interactions. **Table S3.** Starch content and respiration data in various ripening produce.

## Data Availability

All data used in this manuscript is included in the Supplementary Files.

## Abbreviations

AM: Amylose
AP: Amylopectin
AM/AP: Amylose-to-amylopectin ratio
AGPase: ADPglucose pyrophosphorylase
CBM: Carbohydrate binding module
CIS: Cold induced sweetening
GBSS: Granule bound starch synthase
GI: Glycemic index
ISA: Isoamylase
PHO: Starch phosphorylase
PTST: Protein targeting to starch
RS: Resistant starch
SBE: Starch branching enzyme
SS: Starch synthase
StVInv: *Solanum tuberosum* vacuolar invertase
TSS: Total soluble solids
