# Correction: Starch branching enzymes as putative determinants of postharvest quality in horticultural crops

**DOI:** 10.1186/s12870-024-05472-z

**Published:** 2024-08-09

**Authors:** Jingwei Yu, Keyun Wang, Diane M. Beckles

**Affiliations:** 1 Department of Plant Sciences, University of California, One Shields Avenue, Davis, CA 95616 USA; 2 Graduate Group of Horticulture & Agronomy, University of California, Davis, CA 95616 USA; 3 Present address: Institute of Plant and Food Science, Department of Biology, School of Life Sciences, Southern University of Science and Technology, Shenzhen, 518055 PR China

**Correction: BMC Plant Biol 21, 479 (2021)**

**https://doi.org/10.1186/s12870-021-03253-6**

Following the publication of the original article [1], the authors identified in the uploaded version of Fig. 6. During data processing, the authors mislabeled two gene names. The correct figure is given below:

Incorrect Fig. 6

**Fig. 6:**
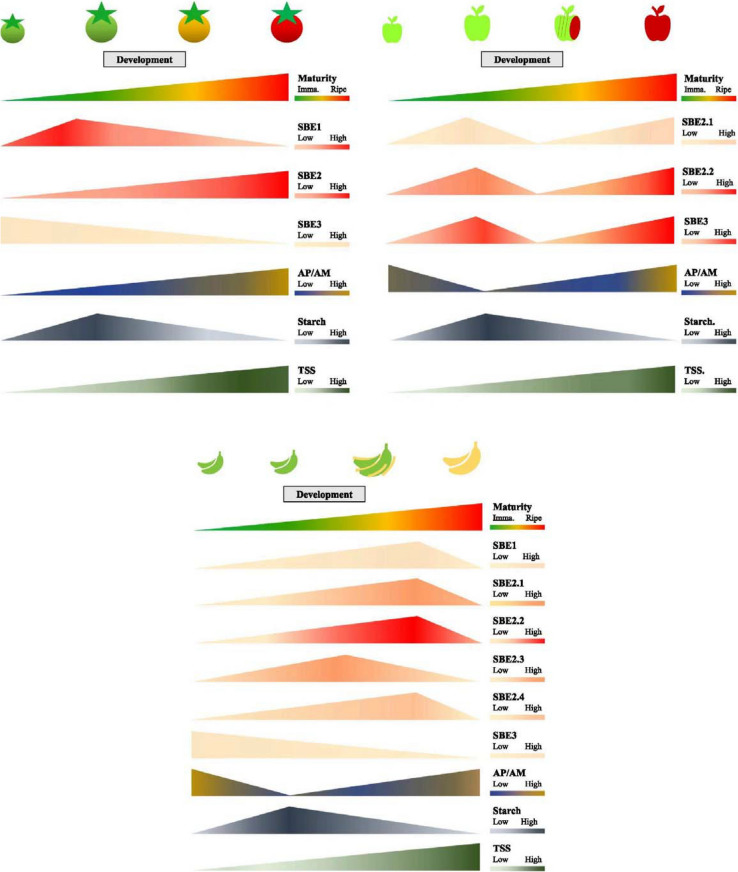
‘Transitory-storage starch’ and relative starch branching enzymes (SBEs) gene expression in developing and ripening fruits. SBE expression patterns in apple difer from that in tomato and banana, in that they distinctly shows bimodal peaks. In addition, unlike the other fruit SBE3s which decrease in expression, the apple SBE3, increases during fruit ripening. The starch content and changes in amylopectin-to-amylose ratio are similar in tomato, apple, and banana. Tomato SBE genes (SlSBE1, Solyc04g082400; SlSBE2, Solyc09g009190; SlSBE3, Solyc07g064830) expressions were obtained from BAR eFP [171], and carbohydrate contents were adapted from [169]. Relative expression level of apple SBE genes (MdSBE2.1, MD12G1020600; MdSBE2.2, MD14G1017700; MdSBE3, MD08G1002300) were retrieved from AppleMDO [172], the starch and sugar data were adapted from two publications [173, 174]. Banana starch and SBEs profles were summarized from three publications [64, 161, 175]. TSS – Total soluble solids. Graphs were drawn in Microsoft® PowerPoint based on published data in Table S1

Correct Fig. 6

**Fig. 6:**
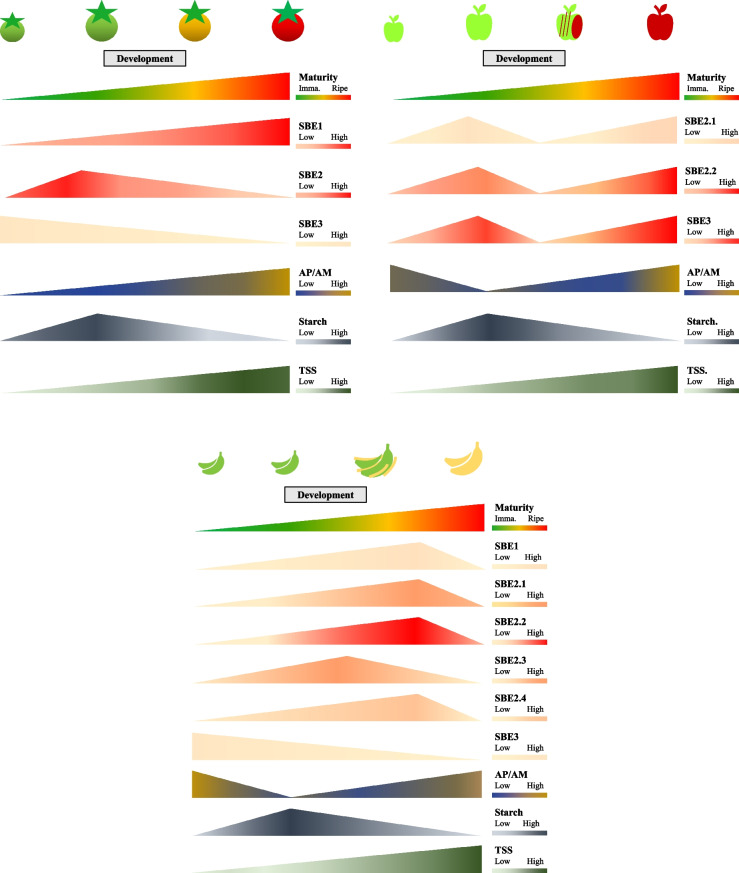
‘Transitory-storage starch’ and relative starch branching enzymes (SBEs) gene expression in developing and ripening fruits. SBE expression patterns in apple difer from that in tomato and banana, in that they distinctly shows bimodal peaks. In addition, unlike the other fruit SBE3s which decrease in expression, the apple SBE3, increases during fruit ripening. The starch content and changes in amylopectin-to-amylose ratio are similar in tomato, apple, and banana. Tomato SBE genes (SlSBE1, Solyc04g082400; SlSBE2, Solyc09g009190; SlSBE3, Solyc07g064830) expressions were obtained from BAR eFP [171], and carbohydrate contents were adapted from [169]. Relative expression level of apple SBE genes (MdSBE2.1, MD12G1020600; MdSBE2.2, MD14G1017700; MdSBE3, MD08G1002300) were retrieved from AppleMDO [172], the starch and sugar data were adapted from two publications [173, 174]. Banana starch and SBEs profles were summarized from three publications [64, 161, 175]. TSS – Total soluble solids. Graphs were drawn in Microsoft® PowerPoint based on published data in Table S1

The original article [1] has been corrected.
