# Supplementary material for: Starch branching enzymes as putative determinants of postharvest quality in horticultural crops
Source: BMC Plant Biol. 2021 Oct 21;21:479. doi: 10.1186/s12870-021-03253-6 (PMC8529802; doi:10.1186/s12870-021-03253-6)
Supplement: Supplementary file 1 — Additional file 1: Figure S1. Mode of action of the starch branching enzymes (SBEs). Figure S2. Starch branching enzyme (SBE) gene structure in select horticultural crops. Figure S3. Protein domain of starch branching enzymes (SBEs) in select horticultural crops and Arabidopsis thaliana. Figure S4. Predicted cis-elements of the 2 Kb upstream region of the SBE coding sequences. Figure S5. Predicted protein-protein interaction ‘STRING’ networks of selected SBE proteins. Figure S6. Correlation between starch content and respiration in diverse ripening produce. [file 12870_2021_3253_MOESM1_ESM.docx]

**Supplementary Material**

**Starch Branching Enzymes as Putative Determinants of Postharvest Quality in Horticultural Crops**

**Jingwei Yu^1,2,†^, Keyun Wang^1^, Diane M Beckles^1,*^**

^1^Department of Plant Sciences, One Shields Avenue, University of California, Davis CA 95616

^2^ Graduate Group of Horticulture & Agronomy, University of California, Davis CA 95616

**^†^**Current address: Institute of Plant and Food Science, Department of Biology, School of Life Sciences, Southern University of Science and Technology, Shenzhen 518055, PR China.

**List of Figures**

**Figure S1**. Mode of action of the starch branching enzymes (SBEs).

**Figure S2**. Starch branching enzyme (SBE) gene structure in select horticultural crops.

**Figure S3**. Protein domains of starch branching enzymes (SBEs) in select horticultural crop and *Arabidopsis* *thaliana*

**Figure S4**. Predicted cis-elements of the 2 Kb upstream region of the SBE coding sequences.

**Figure S5**. Predicted protein-protein interaction ‘STRING’ network of selected SBE proteins.

**Figure S6**. Correlation between starch content and respiration in diverse ripening produce


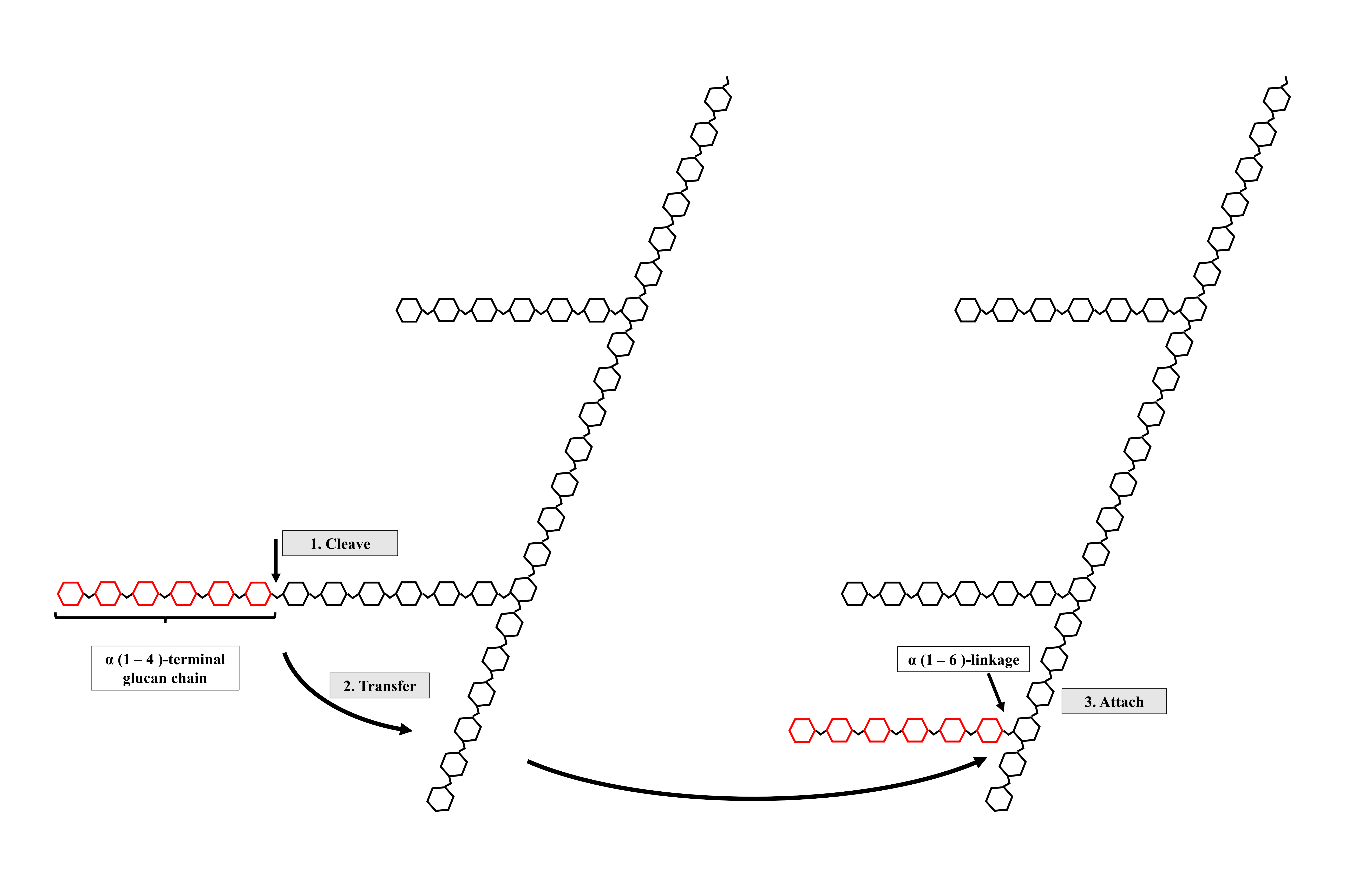


**Figure S1. Mode of action of the Starch Branching Enzymes (SBEs).** SBEs cleave α-1,4-terminal glucan chains, and then they transfer and attach them to another chain, via an α-1,6 glucan linkage. This figure was made by the authors using Microsoft® PowerPoint.


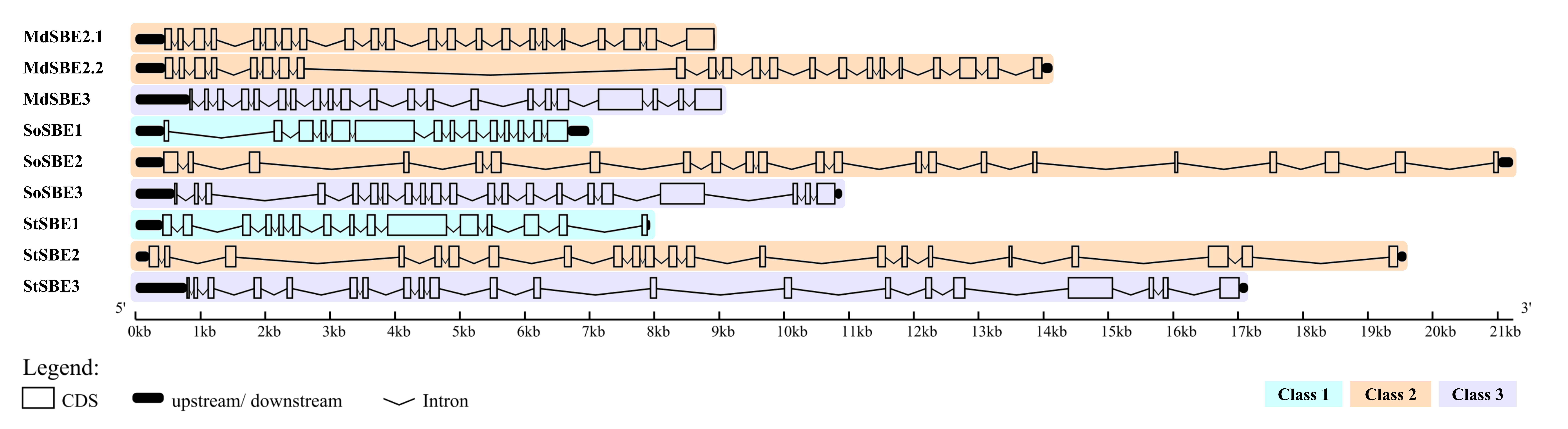


**Figure S2. Starch branching enzyme (SBE) gene structure in select horticultural crops.** SBEs vary in the number of exons (14-22) depending on the species. SBE1, SBE2, and SBE3 commonly have 15, 22, and 21 exons, individually, and the length of coding sequence is around 2600-3100 bp. Exons of ~1 Kb can be seen in SBE1 and SBE3, but only short exons and long introns are found in SBE2. Data was generated using GSDS2.0 [178], by the authors using Microsoft® PowerPoint.


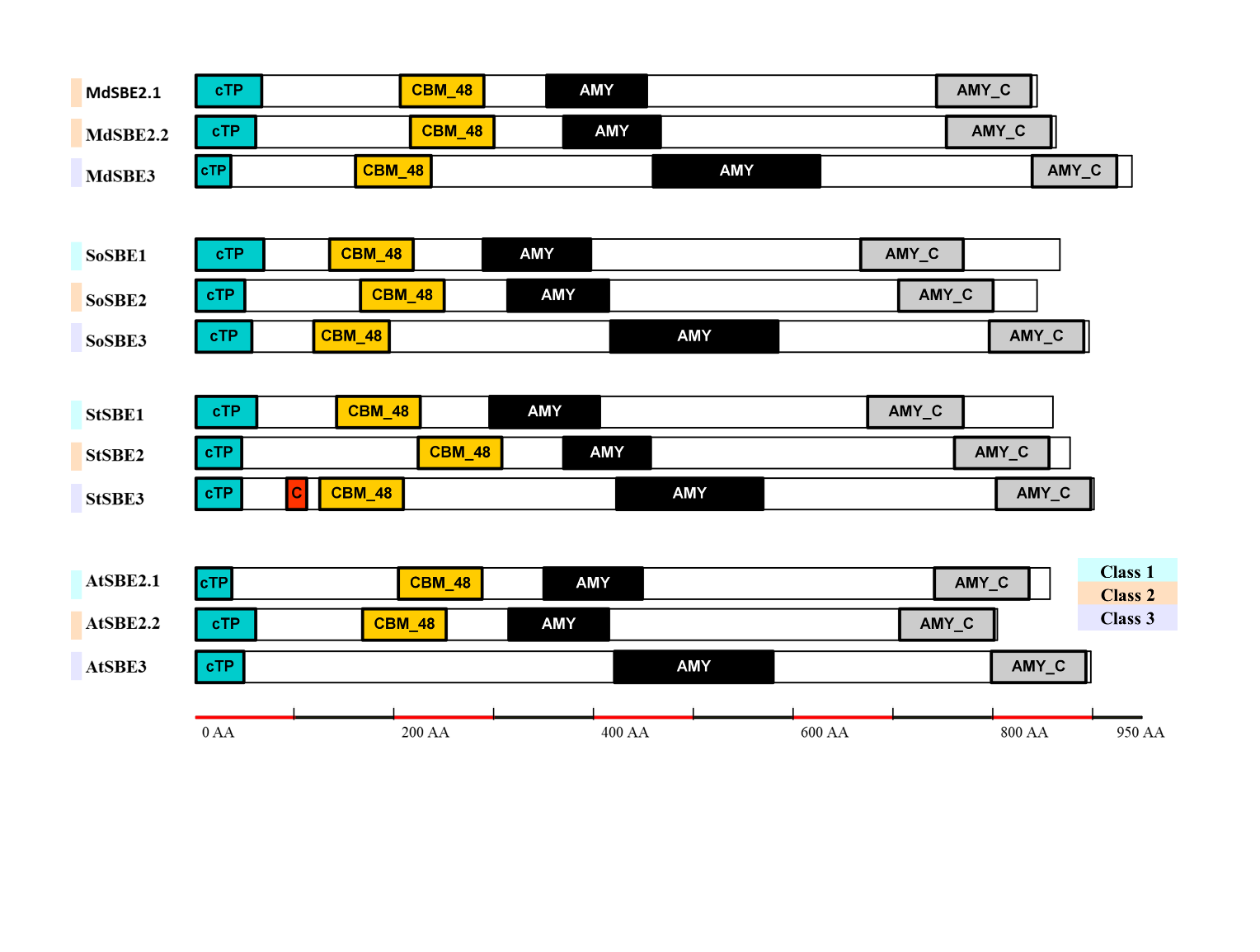


**Figure S3. Protein domain in starch branching enzymes (SBEs) in select horticultural crop and *Arabidopsis* *thaliana*.** The three classes of SBEs all share a similar domain structure: a chloroplast transit peptide (cTP) and a carbohydrate-binding module of the family 48 domain (CBM_48) on the N-terminal of SBE protein; a catalytic, α-amylase family domain (AMY) in the middle; and an α-amylase C terminus (AMY_C) at the C-terminal of SBE. Note that the *Arabidopsis* SBE3 lacks CBM_48 compared to other class 3 SBEs, and the potato SBE3 has a unique coiled-coil motif (C). Species listed include apple (*Malus domestica*), spinach (*Spinacia oleracea*), potato (*Solanum tuberosum*), and *Arabidopsis thaliana*. Transit peptides were predicted using ChloroP [179], and domain features were analyzed with HMMER (Pfam database, <https://www.ebi.ac.uk/Tools/hmmer/>). This figure was made by the authors using Microsoft® PowerPoint.


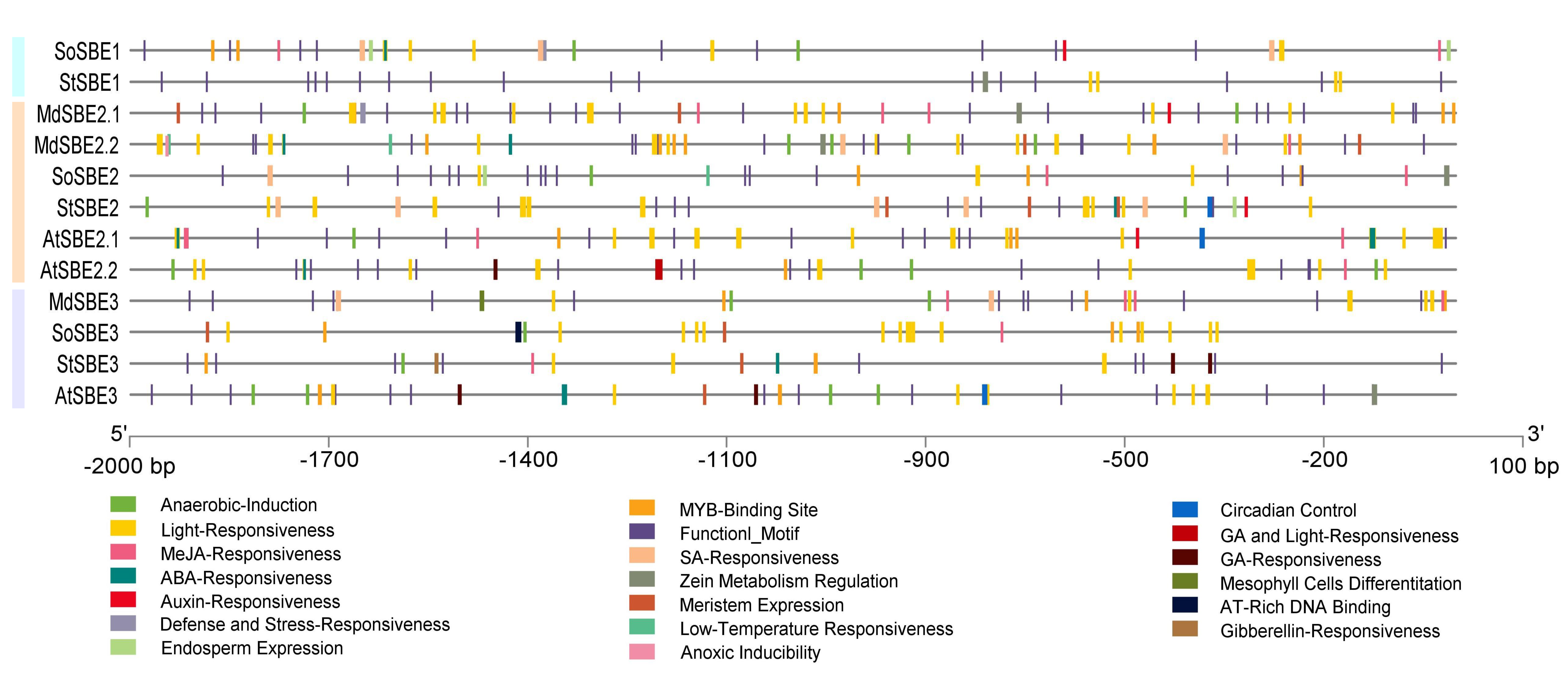


**Figure S4.** **Predicted cis-elements of 2 Kb upstream region of SBE coding sequences.** Around 20 types of cis-element motifs (core promoter motifs were not included) were identified through PlantCARE [180]. Most motifs are hormone (e.g., abscisic acid, ABA; salicylic acid, SA, jasmonic acid, MeJA; and gibberellic acid, GA) and environmental signal responsive, and direct TF binding sites. Species listed include apple (*Malus domestica*), spinach (*Spinacia oleracea*), potato (*Solanum tuberosum*), and *Arabidopsis thaliana*. This figure was generated by TBtools (https://github.com/CJ-Chen/TBtools/releases).


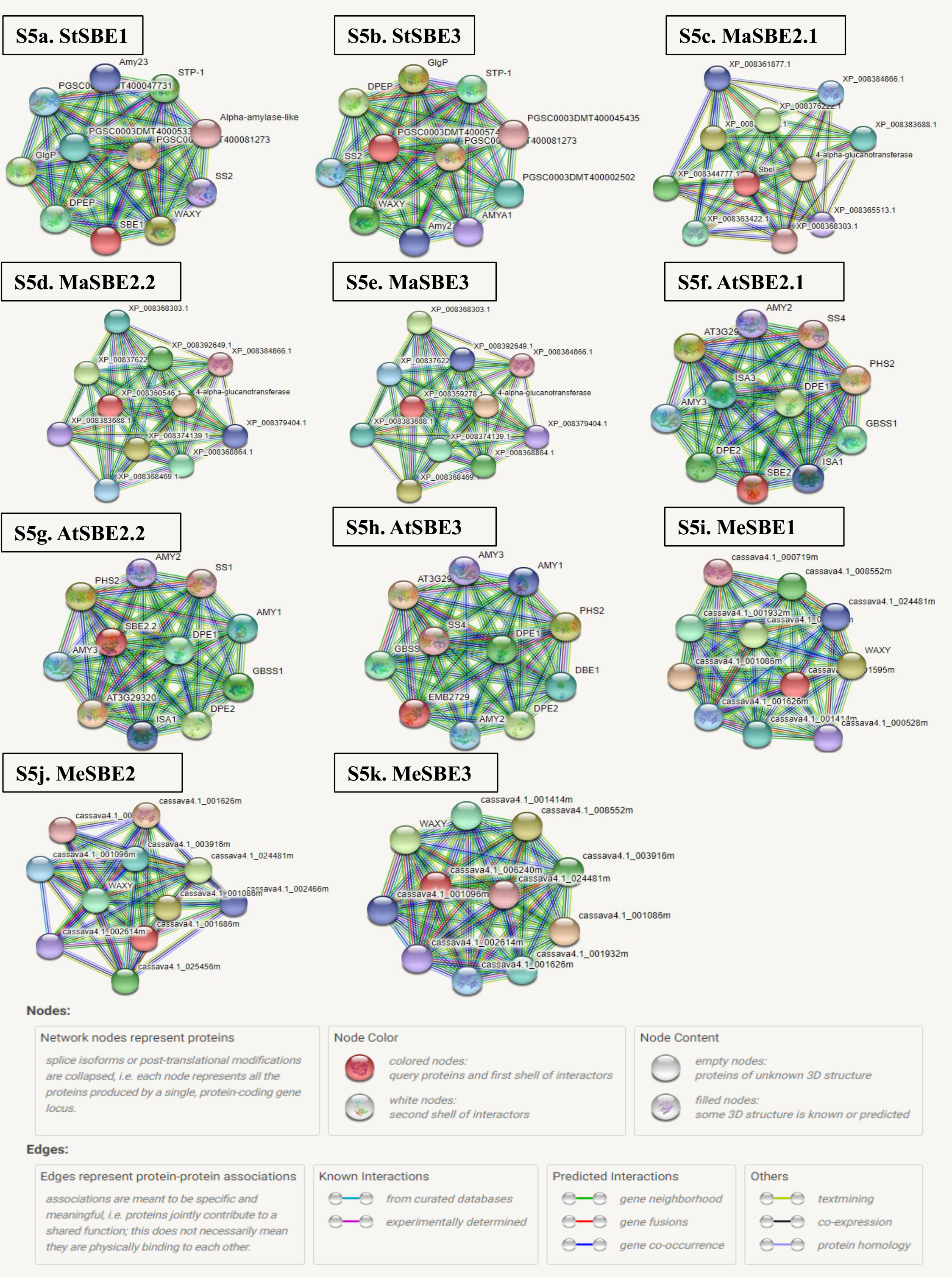


**Figure S5.** **Predicted protein-protein interaction ‘STRING’ network of selected SBE proteins.** a) Potato StSBE1; b) Potato StSBE3; c) Apple MaSBE2.1; d) Apple MaSBE2.2; e) Apple MaSBE3; f) *Arabidopsis* AtSBE2.1; g) *Arabidopsis* AtSBE2.2; h) *Arabidopsis* AtSBE3; i) Cassava MeSBE1; j) Cassava MeSBE2; and k) Cassava MeSBE3. Figures were obtained from STRING (<https://string-db.org>). Key of proteins – AMY – amylase; ISA- Isoamylase; WAXY – GBSS; DBE- debranching enzyme; DPE – disproportionating enzyme; PHS- Starch phosphorylase; DPEP- 4-alpha-glucanotransferase. The raw data used to draw these images, and details of all proteins shown, can be found in the Supplementary Excel File accompanying this manuscript.


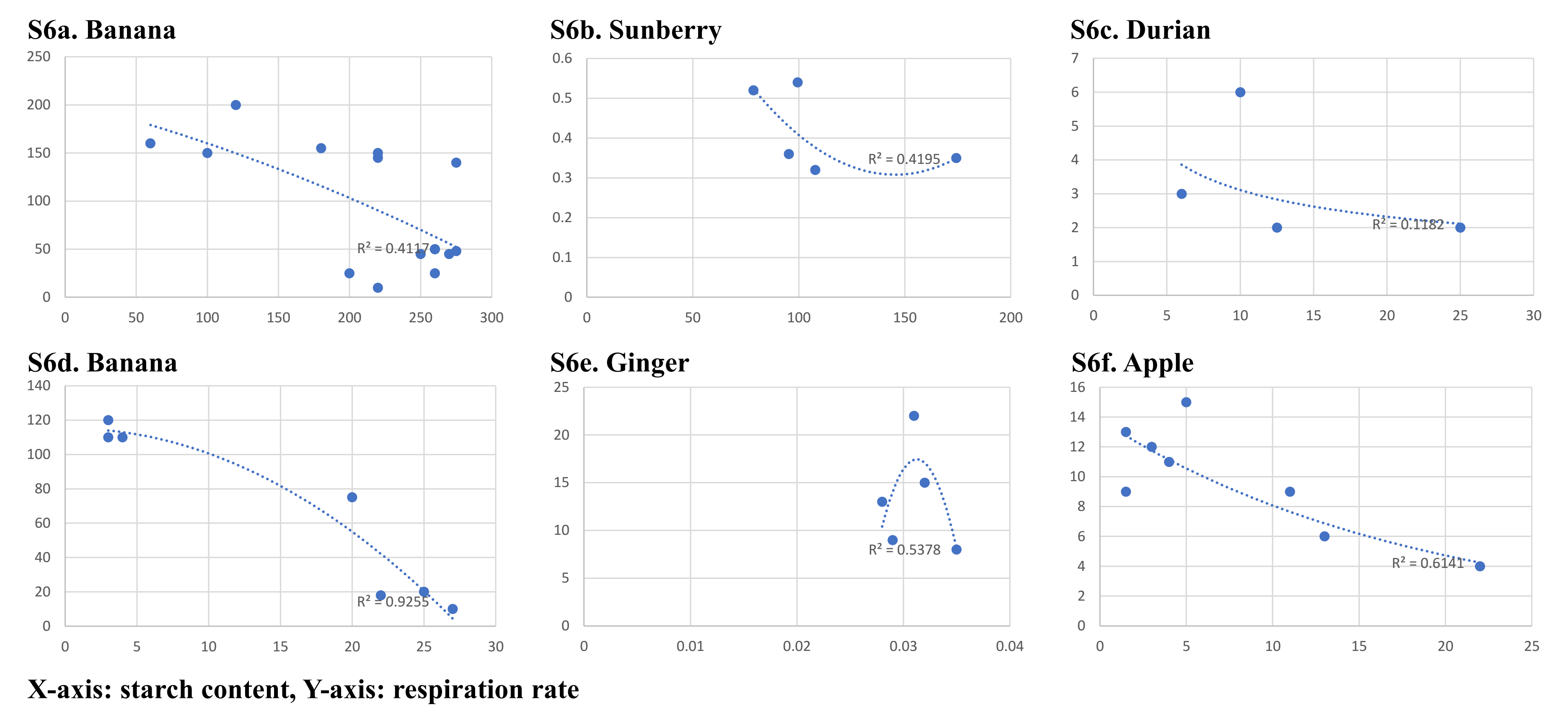


**Figure S6.** **Starch content and respiration correlation in various ripening produce.** a) Banana StSBE1; b) Sunberry; c) Durian; d) Banana; e) Ginger; and f) Apple. The raw data used to draw these graphs can be found in Table S3 of the Supplementary Excel file. Graphs were created by Microsoft® Excel.
